# Taste of Things to Come: Craving Responses to Ingestion of and Mouth Rinse with a Sugary Drink in Connection with Food Cues and Associations with Continuous Interstitial Glucose Measurement in a Healthy Population

**DOI:** 10.3390/nu18010177

**Published:** 2026-01-05

**Authors:** Abdelbare Al Gamode, Rohi Brigid Malik, Joe Butler, Hans-Peter Kubis

**Affiliations:** 1 School of Psychology and Sport Science, Bangor University, Bangor LL57 2PZ, UK; a.algamode@yahoo.com (A.A.G.); rohi.malik@doctors.org.uk (R.B.M.); 2 Faculty of Medical Technology, University of Zawia, Zawia 16418, Libya; 3 School of Psychology, Sunderland University, Sunderland SR1 3SD, UK; joe.butler@sunderland.ac.uk

**Keywords:** food craving, food cues, taste, glucose, sugar, leptin, mouth rinse, soft drink, food intake

## Abstract

**Background/Objectives:** Food cravings are common with high-palatability foods that are high in sugar and/or fat. Food cues can strongly induce food craving, and heightened food cue reactivity is associated with eating disorders and obesity. Sweet taste signalling is suggested to be an important regulator of appetite and food intake, with sensory-metabolic mismatch potentially relevant for the food craving experience. This study investigated the interaction between taste and food cues and food craving in healthy people with and without ingestion of a sugary drink. **Methods:** This study had a randomised crossover design with 47 healthy individuals who participated in two experimental trials. Fasted individuals were exposed to food cues, and food craving pre- and post-exposure was measured via a newly validated method using handgrip force as a response modality. This was followed either by ingestion (ingestion trial) or mouth rinse (mouth rinse trial) of a sugary drink and reassessment of food cue craving responses. Continuous interstitial glucose monitoring was performed using a glucose sensor inserted into the upper arm, and a blood sample for leptin levels was taken. **Results:** A strong food craving response to food cues was bound to the fasted state, while ingestion of a sugary drink blunted food cue reactivity and reduced craving levels. Mouth rinse induced a stable increase in food craving, which reached a maximum after food cues. Interstitial glucose levels over the after-trial periods (incremental area under the curve, iAUC) were significantly higher for the rinse trial day than for the ingestion trial day, which may suggest higher carbohydrate/sugar intake after the rinse trial, while craving levels were associated with iAUC in the rinse trial. **Conclusions:** Outcomes indicate that taste/flavour in connection with food cues may generate an error signal experienced as food craving, whereas receipt of sugars, with concomitant physiological responses, reduces the signal and diminishes food craving. These results highlight the importance of sensory-metabolic mismatch in the food craving experience.

## 1. Introduction

Food craving may be defined as an intense desire or urge towards the consumption of a specific food type, unlike the physiological state of hunger [1,2]. Food craving encompasses various experiences, which include strong urges for seeking and consumption of food, intrusive thoughts and imagery of foods, perception of poor self-control for food consumption, and anticipation of positive and/or negative reinforcement when food is consumed [3,4,5]. Tiffany’s cognitive model of drug use and craving [6] proposed that cravings are based on automatic learned action schemata, which are stimulus-bound and supported by nonautomatic cognitive processes. Consequently, food cravings may be understood as sensory outcomes of automatic processes already running when initiated by food cues and/or emotions and thoughts [7].

Food craving is particularly frequent with highly palatable foods with high energy density containing high levels of sugars and/or fat [8]. While state craving is regularly experienced by people without eating disorders [9,10,11], high trait craving is associated with eating disorder pathology [4,12,13].

Visual experiences and smells can be potent food cues, strongly inducing food craving [14]. However, the reactivity to food cues depends on physiological states, i.e., hunger and satiety [15], and emotional states, i.e., stress [16,17], as well as showing circadian reactivity [18,19]. Moreover, heightened food cue reactivity is associated with eating disorders and obesity [20]. Food cravings are suggested to be strongly connected with the dopamine system [21] in brain areas associated with reward and incentive salience [22].

Reductions in state food craving are generally connected with physiological responses to an increase in nutrient availability in the gastrointestinal tract and concomitant decrease in ghrelin, increase in intestinal hormones (i.e., GLP-1, PYY, CCK) [23], and postabsorptive changes in nutrient levels (i.e., glucose and insulin response) [24] and hormones, i.e., insulin, amylin [25], in the blood. Moreover, vagal responses in connection with pressure/tension changes in the gastrointestinal tract [26], as well as nutrient sensing [27] and metabolic response to absorbed nutrients [28], were reported with craving reduction.

However, the contribution of taste, or more correctly flavour, to the craving response and appetite/satiety is ambiguous [29]. Flavour is thought to be the first signal of receipt of nutrients and could be an early signals that acts as negative feedback to cravings [30], with afferent signals towards various brain areas, including the striatum, insula, hypothalamus, and others [31], in addition to initiating preparatory responses for the ingestion and digestion of food during the cephalic phase [32]. Sweet taste signalling is suggested to be an important regulator of appetite and food intake [31]. Moreover, nutrients also bind to nutrient receptors in the more distal gastrointestinal tract after ingestion [33], using vagal afferents and hormonal release as a response modality [34]. In addition to establishing negative feedback (i.e., glucose-sensing neurons in the hypothalamus [35]), nutrient receptors have also been suggested to play a role in the learning of rewards [36,37]. Palatable foods, as well as foods which are not palatable and need to be digested before receptor-active nutrients are released, should be able to provide information about the energy content of foods and enable reinforcement of rewards/energy content for learning processes [38]. For example, blood glucose levels are shown to impact food-related decision-making in various decision-making tasks [39].

Consequently, it could be hypothesised that flavour/taste signals in the oral cavity might have different functions compared with more distal nutrient sensing and responses. While oral cavity signals are involved in food reward perception and learning, they may also predict energy content related to the taste experience. The more distal gastrointestinal receptors might enable receipt of nutrients, as well as reward/energy learning, with postabsorptive responses further supporting these processes.

The interaction between oro-sensory taste signals and more distal GI nutrient signals may be thought of as a part of a regulatory cycle in which visual cues, together with taste, set predict reward and energy receipt, while ingestion and postabsorptive processes act as an extinction signal for the error signal perceived as cravings. Recent studies have proposed that connection of the former systems could lead to sensory-metabolic mismatch if, for example, non-nutritive sweeteners are used instead of sugars [28,40]. Cogan and Cooper [40] investigated the effects of mouth rinse with sucrose, sucralose, maltodextrin, or water and measured appetite and appetite hormones thereafter, as well as buffet meal energy intake. While energy intake at the buffet meal was not affected by the various mouth rinses, some hormonal responses were specific to the rinse component. However, the researchers did not compare these effects with the effects of ingestion of the rinse components. Mouth rinse with other nutrients, i.e., fatty acids, reduced hunger ratings following a standard meal, suggesting that taste influences hunger perception following the ingestion of food [41]. Moreover, Lauritsen et al. [42] found no influence of oral ingestion of glucose compared with isoglycemic infusion on appetite and satiety, suggesting no contribution of flavour to appetite and satiety perceptions, but did not perform a mouth rinse. Veldhuizen et al. [28] combined tasteless carbohydrates and non-nutritive sweeteners to produce various levels of sweetness with different caloric content and investigated the effects of ingesting these mixtures on fMRI brain responses, liking ratings, and metabolic response. They found that metabolic responses provided a signal for encoding flavour cues with nutritional value in the mesolimbic reward system. Metabolic responses were shown to be related to sweet taste perception. They concluded that non-nutritive sweeteners and sugary beverages might disrupt normal physiological responses to carbohydrate, i.e., sensory–metabolic mismatches. However, in their work, they did not perform mouth rinse trials for comparison. While the literature suggests that there are important interactions between taste and nutrients for the regulation of perceptual modalities guiding eating behaviour, the outcomes do not build a coherent picture of the mechanisms involved.

Based on the current literature, more work is needed to understand the effect of flavour/taste and food cues on food cravings, with and without GI receipt of nutrients.

Food cravings can be assessed by various questionnaires [43]. In this study, we used the Food Craving Questionnaire State (FCQ-S) [3] to assess state food craving. The questionnaire measures several dimensions of food craving: in-the-moment intense desire to consume food, outcome expectancies of positive reinforcement and negative reinforcement from eating, anticipated lack of control over eating, and physiological states that may trigger craving. The construct validity of the FCQ-S was shown in connection with expected score alterations pre- and post-food intake [3,44]. However, the use of Likert scales can potentially produce response bias due to the design of the scale, like central tendency bias and others [45]. Therefore, we used handgrip force as a surrogate response measure for the FCQ-S statements. Handgrip forces were measured with a dynamometer in connection with FCQ-S statements, where the level of force applied expressed the level of agreement with the questionnaire statements. Validation of the measurement method and comparison with the Likert scale responses are reported in the Supplementary File. However, outcomes show that the use of handgrip force is a valid and more sensitive measure of FCQ-S responses compared with the Likert scale (see Supplementary File).

To investigate the interaction between taste and food cues and food craving, we used a randomised crossover design. Forty-seven healthy individuals participated in two experimental trials. During the first trial, fasted individuals were exposed to visual food cues and reported their level of food craving before and after the exposure to food cues, followed by ingestion of a sugary drink and reassessment of craving levels. The second trial consisted of the same process; however, instead of ingesting the sugary drink, but the individuals repeatedly rinsed their mouths with the sugary drink without ingestion. Continuous interstitial glucose monitoring over 14 days was performed with the inclusion of the two experimental trials. A blood sample for leptin measurement was taken, due to the major importance of leptin for energy balance regulation [46,47] and influencing the taste response in the hypothalamus [30]. Further, a battery of psychological questionnaires regarding eating behaviour, craving, stress, and body characteristics was administered.

We hypothesised that food cues would increase craving levels in the fasted state, while taste and ingestion of a sugary drink would reduce craving levels and blunt the response to food cues.

Secondly, we hypothesised that the mouth rinse would not reduce food craving, but would maintain craving at high levels with and without food cues, reasoning that the taste experience together with food cues would set up an error signal for nutrient receipt and energy absorption/metabolism, which would be experienced as food cravings.

Thirdly, we expected that the incremental area under the curve (iAUC) of continuous glucose measurements in the daytime period after the trials would show that mouth rinse and concomitant increase in food cravings would lead to a higher iAUC compared to the iAUC of the period after the ingestion trial. This was suggested by the assumption that elevated food craving induced by the mouth rinse trial would drive sugar/carbohydrate intake in the period following the trial beyond the level of the ingestion trial, where the error signal of craving was extinct by the receipt, physiological response, and metabolism of sugar.

## 2. Methods

### 2.1. Ethical Approval

This study was ethically approved by the Ethics Committee of School of Psychology and Sport Sciences at Bangor University, ethics number: 2023-17333.

### 2.2. Participants

Using G*Power 3.1 for calculating a priori sample size, we used the outcomes of our validation study (Supplementary File) to calculate the effect size for food craving alteration in response to food intake (*f* = 0.48). However, due to the potentially lesser effects of sugary drink intake on craving, we used an effect size of *f* = 0.35. With an alpha level of 0.05 and 95% power, based on a repeated measures ANOVA within-between interaction, we calculated a sample size of at least n = 40. Forty-seven healthy participants (17 males and 30 females) were recruited from Bangor University and the local community in the Bangor area. Recruitment was performed via advertisements in the university, local sports clubs, and student Facebook groups. The inclusion criteria were as follows: age 18 to 60 years, injury-free, not pregnant, non-smoker, healthy, i.e., without any cardiovascular, metabolic, or pulmonary disease, BMI between 18 and 35 kg/m^2^, no medications which might limit participation, no vegetarians/vegans due to food image selection, and no eating disorders. Health-screening questions were sent via a Qualtrics survey link to check eligibility for taking part. All study information was sent and explained verbally, with the opportunity to ask questions on the first visit, and consent was taken. Participants received a reimbursement of £100 for their time after successful completion of all sessions.

### 2.3. Procedures

The study included four testing sessions conducted in the laboratories of the School of Psychology and Sport Sciences. The study material was integrated into Qualtrics (www.qualtrics.com). Sessions 1 and 4 were performed on the first and the final days of two weeks, while sessions 2 and 3 were performed on separate mornings within the 2 weeks, at least 3 days apart.

Session 1: participants performed this session after an overnight fast, having refrained from strenuous exercise and alcohol in the preceding 24 h. The session consisted of the following sections:

- Body characteristics were assessed, including sex, age, height, weight, and body composition (i.e., body fat percentage).
- Participants were asked to fill in self-report questionnaires (IPAQ, FCQ-T, TFEQ, PSS, PANAS, see Measurements).
- Fasting earlobe blood samples were collected to measure blood glucose levels and for analysis of leptin levels (see Measurement for details).
- Familiarisation with the handgrip force measure as a measure of agreement with the statements on the Food Craving Questionnaire State (FCQ-S). The procedure was explained, and a test trial was performed, as well as three maximum handgrip forces for standardisation of cravings (see validation study in Supplementary Information).
- The participants were asked to rate their current cravings (FCQ-S statements on the computer screen) by applying handgrip forces for perceived agreement with statements. The handgrip dynamometer was connected to an A/D converter (PowerLab system) to record the handgrip forces (no visual feedback was available for the forces applied).
- Participants were shown neutral images (household items) while being asked to perform a cognitive sorting task to focus attention on the images (see Measurement for details). After the neutral cue exposure, the assessment of their current cravings was repeated.
- Participants consumed 250 mL of a commercially available orange-flavoured cordial containing 50 g of sugar and then rested for 25 min.
- During the resting period, a continuous glucose monitoring sensor (CGMS) was attached to the back of the upper arm (see Measurement for details).
- Participants performed the 3 min step test to assess cardiovascular fitness (see Measurements).

Session 2 and 3—the second and third sessions (ingestion trial or rinse trial)—were performed in a counterbalanced randomised manner, and each session was conducted on a separate day (at least 72 h apart) within the 2-week CGM measuring period. Participants performed the sessions after an overnight fast, having refrained from strenuous exercise and alcohol in the preceding 24 h. All steps of the sessions were timed by the Qualtrics program, with images and FCQ-S items embedded in the program.

**Figure 1:**
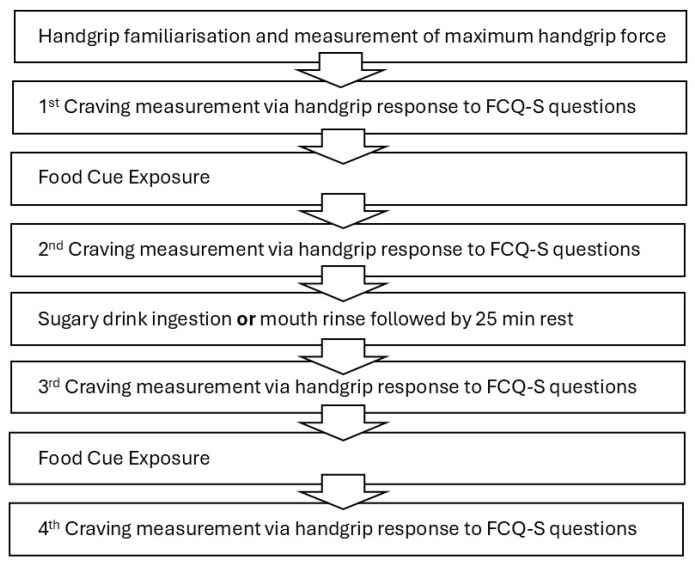
Session 2 and 3 protocol. FCQ-S: Food Craving Questionnaire—State.

Participants produced three maximum handgrip forces with the dynamometer for individual standardisation of the craving responses. Participants were asked to rate their current cravings (FCQT-S questions) by applying handgrip forces for perceived agreement with statements (pre-cue measure). The researcher scanned the glucose sensor both before and following the craving ratings. Subsequently, participants were shown a set of 15 images, including 11 sweet and 4 savoury food images (cues), on a computer screen. The images were shown twice to the participants for 6 s each in a random order over 3 min while being asked to perform a cognitive task to focus attention on the images (see Measurement for details). After the cue exposure, the assessment of their current cravings was repeated (post-cue measure), and glucose levels were scanned again. This was followed by the participants either ingesting the orange cordial drink (Belvoir Farm) containing 50 g of sugar in 250 mL of water (ingestion Trial) or rinsing their mouth with the same volume of cordial (rinse Trial). Following both ingestion and rinse, participants flushed their mouths with water to avoid continued taste effects. After the mouth rinse in the rinse trial, participants ingested 250 mL of water to match the ingested volume in the ingestion trial. Then, participants rested for 25 min; during this resting period, the glucose sensor was scanned every 5 min. After the resting period, craving assessments were repeated, and glucose levels were recorded (pre-cue measure). Concurrently, the exposure to food cues was repeated, and a further craving assessment was performed with glucose measures recorded (post-cue measure). A flowchart of the session procedures is presented in Figure 1.

Session 4: This session was performed at the end of the 2 weeks of continuous glucose measurement. After removing each participant’s CGM, the collected data were downloaded, and the participant was debriefed.

### 2.4. Measurements

#### 2.4.1. Demographic and Body Characteristics

Sex, age, height (standard stadiometer), weight, and body composition (i.e., body fat percentage) were measured via bioelectrical impedance using the Tanita BC-418 MA system.

#### 2.4.2. Self-Report Measures

Participants were asked to fill in the following questionnaires during the first session:

- The International Physical Activity Questionnaire (IPAQ) [48] is a validated instrument for measuring regular physical activity. Reliability was assessed in 12 countries [49], with Spearman’s rho 0.81.
- The FCQ-T [3] assesses food cravings via 39 items that address behavioural, cognitive, and physiological components of cravings. The FCQ-T has an overall CRα of 0.98, with subscale alphas ranging from 0.71 to 0.95 [3]. Total scores range from 39 to 234, with higher scores indicating greater trait food craving.
- The Food Cravings Questionnaire-State (FCQ-S) assesses the strength of food cravings that are influenced by one’s current state. It uses 15 items measuring agreement with statements connected to five dimensions (see Measurements of food craving via handgrip dynamometer). The FCQ-S has an overall CRα of 0.96 [3].
- The Three-Factor Eating Questionnaire (TFEQ) [50] consists of 18 items evaluating three dimensions of eating behaviour: ‘uncontrolled eating’, ‘cognitive restraint’, and ‘emotional eating’, with Cronbach’s alpha for these scales being above 0.70 [51]. Subscales were transformed to 0 to 100 scales, with high scores indicating greater expression of each eating behaviour.
- The Positive and Negative Affect Schedule (PANAS) [52] assesses affect using two separate scales for negative and positive mood scales; CRα: 0.89 [53]. Scores range from 10 to 50, with higher scores indicating greater positive or negative affect, respectively.
- The Perceived Stress Scale (PSS) assesses individuals’ appraisal of stressful situations in their lives [54]. Participants respond to 10 items on a Likert scale ranging from 0 = ‘Never’ to 4 = ‘Very Often’ to assess unpredictability, lack of control, and pressure in their lives over the past month, with Cronbach’s alpha for this scale above 0.70 [55]. Scores range between 0 and 40, with higher scores indicating greater perceived stress. Scores higher than 27 are considered high perceived stress.

#### 2.4.3. Continuous Glucose Monitoring (CGM)

Continuous glucose monitoring (CGM) involves wearing a subdermal sensor that automatically monitors and tracks interstitial glucose levels continuously for up to 14 days. The CGM sensor (Abbott FreeStyle Libre 2 sensor, Abbott House, Maidenhead, England) measures interstitial glucose levels at 1-min intervals and transmits the data to a monitoring device (i.e., mobile phone). Over 2 weeks, interstitial glucose levels were continuously monitored after attachment to the back of the upper arm of participants using the applicator provided with the sensor. Upon applying the sensor, participants downloaded the FreeStyle LibreLink 2.8.4 mobile application on their smartphones and registered for the LibreView 3.2.2 online diabetes management system. Participants were instructed on how to use their phones to scan the sensor regularly. Participants were not encouraged to monitor their glucose levels and were informed that the CGM was used to avoid taking blood samples during experiments in the lab; they were informed to continue their usual diet habits during the 14 days. Glucose levels were measured in mmol/L, and glucose variability was automatically assessed by calculating the percent coefficient of variation (%CV), providing a measure of glucose fluctuation over time. The data were automatically uploaded to LibreView every time the participant scanned the FreeStyle Libre 2 sensor. The participants were asked to scan the sensor every 8 h during the day over the study period to ensure data were uploaded to LibreView. Subsequently, data were downloaded in the last session. In addition, CGM data were collected during the trials to monitor alterations in interstitial glucose. Data were continuously recorded, and the delay to reassessment of cravings was 25 min in both trials after ingestion and mouth rinse, respectively.

#### 2.4.4. Calculation of the Incremental Area Under the Curve (iAUC) of Glucose Levels over Time

The iAUC of post-trial glucose was calculated to investigate the effect of trial type on glucose response. The iAUC was determined by summing the changes in glucose levels relative to the baseline fasting reading over 10 h, starting 2 h after each trial. This approach, which aligns with the method described by Matthan et al. [56], focuses on incremental changes in glucose levels above the baseline value, considering only increases in glucose concentrations.

#### 2.4.5. Cardiovascular Fitness Test

Estimation of cardiovascular fitness (VO_2_MAX; mL/kg/min) was performed using the validated Tecumseh Test (3 min step test) with a 20.3 cm stepper [57]. The test consists of an initial 2 min resting phase while the participant is seated; then, after exactly 2 min, the participant started stepping up and down (step up-up-down-down) from the stepper at a rate of 96 beats per minute (the rhythm was given by a metronome) for 3 min, followed by an immediate stop, sitting down, and remaining seated with the arms by the sides and the legs uncrossed for 1 min. Heart rate data were recorded automatically throughout the test via a V800 Polar heart rate monitor; the heart rate data were used to estimate each participant’s cardiovascular fitness level based on an equation using the number of heartbeats counted from 30 s to 60 s post-test (HB30to60) (1 min recovery period).

#### 2.4.6. Cue Exposure

Cue exposures were performed with selected food images (eleven sweet food images and four savoury food images) during the ingestion and rinse trials or household items images (session 1). Food images were formerly tested for recognisability and appealing features using a group of volunteers. For each cue exposure, 15 images were shown to the participants for 6 s each, and images were repeated in randomised order over 3 min. To ensure that participants focus on the images, a cognitive task was embedded in the cue exposure. There were two types of task: the first variation used food images (sweet and savoury), and the second variation used everyday household objects to not arouse food cravings, as a control. The cognitive tasks, being part of the cue exposures, displayed images with word attributes (‘sweet’, ‘savoury’ for food images and ‘round’, ‘edgy’ for household items) Five to nine incorrect pairings were embedded in the 30 image exposures. Participants were instructed to identify and count the incorrect pairings of images and words. The purpose of the test was to focus attention on images for stimulation of cravings through food cue exposure.

#### 2.4.7. Blood Sampling

Fasting blood samples were collected from the earlobe to measure fasting blood glucose levels and leptin levels. Blood samples (250 µL) were collected into two capillary blood tubes (Microvette^®^ APT 250 EDTA K2E, 250 µL). Fasting glucose was analysed using a HemoCue^®^ Glucose 201+ Analyser (HemoCue^®^ AB, Ängelholm, Sweden). Blood was then centrifuged at 3000 rpm for 10 min at 4 °C (Eppendorf Centrifuge 5810 R; Eppendorf SE, Hamburg, Germany), and the plasma was harvested and stored at −80 °C for later analysis. Leptin concentrations were determined via enzyme-linked immunosorbent assay (ELISA) (BioVendor Research and Diagnostic Products, BioVendor–Laboratorni medicina a.s., Brno, Czech Republic) using a plate reader (Fluostar Omega, BMG Labtech, Ortenberg, Germany). All samples were analysed in duplicate. The intra-assay coefficient of variation for leptin was 8%.

#### 2.4.8. Measurements of Food Craving via Handgrip Dynamometer-Food Craving Questionnaire-S

In this study, handgrip force was used to assess the level of agreement with the statements on the Food Craving Questionnaire State (FCQ-S) [3] to assess cravings, instead of the Likert scale. The method was validated in 32 healthy participants (see Supplementary File). The handgrip dynamometer used to assess craving levels was connected to an A/D converter (PowerLab system) to record handgrip forces. The craving response, as measured by handgrip force (HG), was standardised using the formula (HG value–minimum HG value)/(maximum HG value–minimum HG value), with the maximum being the highest value of the three maximum HG forces produced at the start of the sessions. The minimum value corresponded to the baseline, defined as holding the dynamometer without voluntary force production. The participants were instructed to perform three maximum handgrip forces before each session to be used for standardisation.

### 2.5. Statistical Analyses

All statistical analyses were performed using IBM SPSS Statistics 29 and Microsoft Excel 2010. All data are reported as means ± SD, unless otherwise stated. Statistical significance was set at *p* < 0.05. All data were assessed for relevant assumptions: normality, outliers, and homogeneity of covariance. Food craving and interstitial glucose responses were analysed using repeated-measures ANOVA. The study employed a randomised, counterbalanced crossover design in which all participants completed both experimental visits (sugary drink ingestion and mouth rinse control). Each participant was treated as the repeated subject factor to account for within-subject dependency. Within-subject factors included visit (ingestion vs. mouth rinse), physiological state (fasted vs. postprandial), and cue exposure (pre- vs. post-food cues). Where significant effects were detected, post-hoc within-subject contrasts were performed using paired t-tests with Bonferroni correction. Incremental area under the curve (iAUC) values were compared between conditions using paired *t*-tests. Spearman’s correlations and Pearson’s partial correlation were used to analyse relationships between variables.

## 3. Results

### 3.1. Body Characteristics, Blood Parameters, and Self-Report Measures

In total, 47 participants completed the study. Body characteristics, self-report measures, and fasting blood levels of glucose and leptin are presented in Table 1 and Table 2.

Self-report questionnaires showed that participants had moderate cravings (FCQ-T: 120.38 ± 30.55), and the PANAS-PA and PANAS-NA yielded moderate scores for both positive and negative affects. The mean Perceived Stress Scale (PSS-10) score was 31.64 ± 3.76, indicating a high level of perceived stress among participants. TFEQ scores indicate moderate levels of cognitive restraint (53.84 ± 10.86), uncontrolled eating (48.86 ± 13.46), and emotional eating (58.39 ± 26.16).

The mean fasting glucose level was 4.63 ± 0.47 mmol/L, revealing healthy fasting levels throughout. The participants had a normal range of fasting leptin levels, with a mean of 9.77 ± 9.75 ng/mL (Table 2).

### 3.2. Interstitial Glucose Levels

The CGM data over the 14-day period revealed that participants had a mean glucose level of 5.58 mmol/L, a daytime glucose mean of 5.72 mmol/L, and a nighttime glucose mean of 5.31 mmol/L, as well as a mean glucose variability of 16.06%CV. The data revealed normal glucose homeostasis in our sample (Table 3).

### 3.3. Changes in Interstitial Glucose Levels

**Figure 2:**
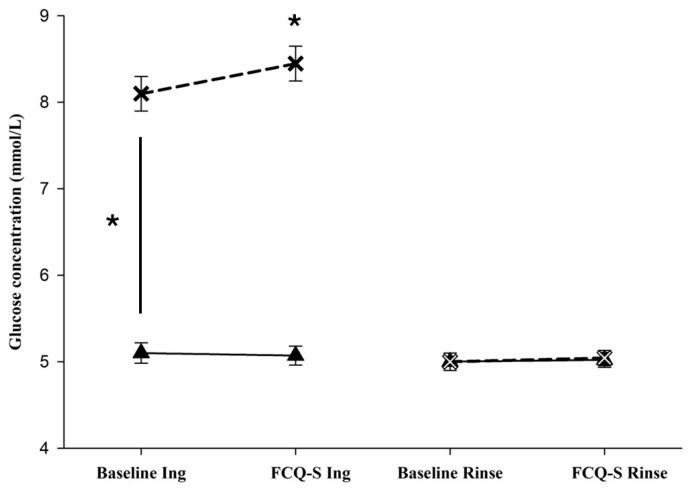
Changes in glucose levels during the ingestion trial (Ing) and rinse trial (Rinse); * indicates a significant change in glucose levels *p* < 0.05; solid lines with triangle symbols represent fasted state; dashed lines with X symbols represent after intake/rinse states; Baseline Ing/Rinse: at baseline conditions in the respective trial; FCQ-S Ing/Rinse: during FCQ-S measurements after ingestion or rinse.

We hypothesised that interstitial glucose concentrations would be only elevated by ingestion of the sugary drink (ingestion trial) and not during the rinse trial. The repeated measures ANOVA showed a significant main effect of time on glucose levels (*F*(1, 184) = 45.150, *p* < 0.001), with a significant interaction between the time factor and trial type (*F*(1, 184) = 33.763, *p* < 0.001) and between the time factor and the state (fasted vs. after intake) (*F*(1, 184) = 46.830, *p* < 0.001). Glucose levels were higher after ingestion of the sugary drink (*F*(1, 184) = 91.972, *p* < 0.001). Pairwise comparison (paired *t*-test) indicated that glucose levels increased significantly only during the ingestion trial after sugary drink intake (Table 4, Figure 2). Moreover, no significant differences were detected during the rinse trial in both states (fasted and after rinse). In summary, the interstitial glucose levels were significantly increased after sugary drink intake in the ingestion trial only and remained at baseline levels during the rinse trial in both states (fasted and after rinse).

Furthermore, we expected a significantly higher area under the curve (AUC) for interstitial glucose levels after the rinse trial compared with the ingestion trial. CGM data were analysed on the trial days between 2 h after the specific trial and the following 10 h. The area under the curve (AUC) of all glucose measures during these periods was calculated (see Methods). The interstitial glucose AUCs after the ingestion and rinse trials had means of 28.98 mmol × time and 38.56 mmol × time, respectively (Table 5).

The mean iAUC after the rinse trial was significantly higher than the mean iAUC after the ingestion trial (*t*(46) = −2.317, *p* = 0.025), showing that glucose levels over the analysed time period were significantly higher for the rinse trial day than for the ingestion trial day.

### 3.4. Changes in Craving Scores

**Figure 3:**
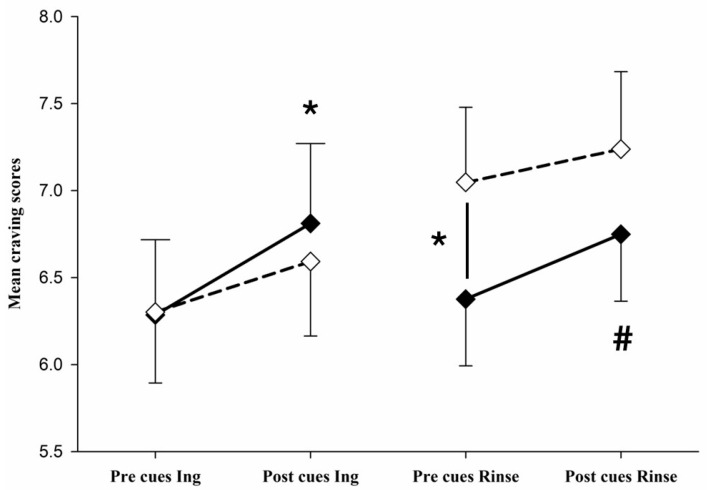
Changes in food craving scores during the ingestion trial (Ing) and rinse trial (Rinse); * indicates a significant change in craving *p* < 0.05; # indicates a trend towards significant change in craving *p* < 0.10; the figure shows means and SEs; solid lines with filled boxes represent the fasted state; dashed lines with open boxes represent after intake/rinse state.

We hypothesised that craving responses to food cues would be particularly strong in the fasted state, with a significant increase in cravings after exposure to food cues, and that the effects of food cues would decline after ingestion of a sugary drink (ingestion trial). In addition, we hypothesised that mouth rinse without ingestion of sugar would lead to a maintenance of cravings levels at high levels after the rinse, and therefore with smaller effects of food cues on craving due to elevated levels. Craving data were analysed by mixed model ANOVA with repeated measures, revealing a significant main effect of time on craving intensity (*F*(1, 184) = 11.720, *p* < 0.001), without significant interactions. Follow-up tests (paired t-test) revealed that, in the fasted state, there was an increase in craving intensity from pre- to post-cue exposure in both trials (*t*(46) = −0.57, *p* = 0.007, ingestion trial and *t*(46) = −1.98, *p* = 0.054, rinse trial), showing that food cues were effective in the fasted state. However, after ingestion and after mouth rinse, no significant alterations in cravings were detected after food cue exposure. Moreover, while ingestion of sugar reduced food craving towards pre-cue fasting levels (Table 6, Figure 3), mouth rinse elevated the craving levels, which were maintained throughout the cue exposure period (pre-cue craving fasted versus pre-cue craving after rinse (*t*(46) = −2.90, *p* = 0.006) (Table 6, Figure 3). The results revealed that, unlike sugar ingestion, which led to a reduction of craving and blunting of food cue response, mouth rinse induced a stable increase in craving.

Furthermore, participants were exposed to neutral cues (household items) during the first session as a control for the sweet food cue effects. Changes in craving scores were assessed before 6.11 ± 2.66 and after 6.52 ± 3.14 exposure to neutral cues in a fasted state. Craving scores pre-cues and after exposure to neutral cues showed a marginal, non-significant effect (*t*(46) = −1.746, *p* = 0.087).

In summary, a strong food craving response to food cues is bound to the fasted state, while differential effects of the ingestion of sugar and mouth rinse were detected. Mouth rinse induced a stable increase in craving, while ingestion of sugar reduced cravings towards pre-cue levels.

### 3.5. Correlation Analysis

To further investigate the influence of participants’ characteristics on their responses, such as changes in craving and glucose levels, correlation analyses were performed. The aim was to explore the potential role of factors like body composition, cardiovascular fitness, and hormonal levels on the observed craving and glucose responses within the experimental design.

Spearman’s correlation analysis revealed that craving change (difference in craving between pre- and post-cue exposure) was negatively correlated with body fat % *(r* = −0.371, *p* = 0.010) and BMI (*r* = −0.426, *p* = 0.003) in the fasted state, suggesting that participants with lower body fat and BMI experienced higher craving responses to sweet food cues when fasted (Table 7). Significant associations were not observed after ingestion or rinse.

In addition, leptin levels were significantly positively correlated with body fat % (*r* = 0.796, *p* < 0.001), showing the expected association. Craving levels post food cue exposure in the fasted state were negatively correlated with leptin levels (*r* = −0.345, *p* = 0.017). The findings indicate that individuals with lower leptin levels, lower body fat, and lower BMI show a stronger craving response following cue exposure when fasted. However, a small positive correlation was observed between leptin levels and FCQ-T scores (*r* = 0.325, *p* = 0.026), indicating that people with higher leptin levels tended to have higher food-craving traits.

Additionally, only after the rinse trial, the area under the curve (iAUC) had a positive significant correlation with glucose variability (*r* = 0.488, *p* < 0.001), showing that, the higher the glucose variability, the higher the iAUC after the rinse trial (Table 6). iAUC after mouth rinse was positively correlated (controlling for sex) with craving levels after cue exposure (*r* = 0.321, *p* = 0.03), suggesting an influence of craving levels on carbohydrate/sugar intake after mouth rinse. An association of iAUC with craving levels was not seen in the ingestion trial. However, partial correlation, controlling for sex, revealed that iAUC after the ingestion trial was negatively correlated with leptin (*r* = −0.369, *p* = 0.012) and BMI (*r* = −0.404, *p* = 0.005) (Table 6), suggesting that intake of carbohydrate/sugar was generally moderated by leptin when sugar was formerly ingested in the ingestion trial. The outcomes suggest that craving levels influenced food intake when craving levels remained high (rinse trial), while leptin moderated intake when sugar was formerly ingested (ingestion trial).

## 4. Discussion

This study investigated the influence of sugar ingestion and mouth rinse on sweet food cue-induced food cravings in a healthy, young sample population. Interstitial glucose was measured by continuous glucose monitoring using glucose sensor technology, and sweet food cravings were measured with a new validated method using handgrip forces as an alternative measure for craving intensity. Food cue-evoked cravings were diminished by ingestion of sugar, with a concomitant increase in interstitial glucose. In contrast, food cues in connection with mouth rinse maintained elevated food cravings before and after further food cue exposure. Mouth rinse-induced food cravings were associated with a significantly higher iAUC of interstitial glucose measures over the day after the rinse trial compared with the ingestion trial. The effect on food cravings levels was moderated by leptin levels and body characteristics.

### 4.1. Physiological and Food Craving Responses to Ingestion and Mouth Rinse

Our study shows that food cues are particularly effective in the fasted state for increasing food cravings, in agreement with former studies [58,59]. We focused on sweet food cues to enable a specific connection between food cues and manipulations with a sugary drink and mouth rinse. Ingestion of sugar reduced cravings and craving response to food cues significantly, suggesting that post-ingestive nutrient signals and concomitant hormonal changes not only provided negative feedback for modifying state craving levels, but also reduced reactivity to food cues [15]. Food craving is a multidimensional construct which encompasses physiological and learned components affecting appetite, motivation, and emotions [60]. Visual cues, smells, and taste are known to initiate feedforward responses during the cephalic phase, including ghrelin secretion in the stomach [61], as well as increasing motivation for food intake and emotional responses, which manifest as appetite/hunger and cravings [62]. Mesolimbic pathways are known to receive input from visual, oro-sensory, and olfactory stimuli, modifying the dopaminergic system linked to food wanting, food reward prediction, and emotional arousal (i.e., incentive salience) [22,63], and signals are integrated into the hypothalamic areas for regulation of food intake. The principal physiological regulation cycles also include negative feedback to the hypothalamic areas, i.e., AgRP and POMC neurons [64]. These areas express receptors for various nutrients, i.e., glucose, as well as numerous hormones like insulin, leptin, and various gut hormones, but vagal afferents are also involved in the regulation of hypothalamic modulation [65]. Vagal afferents are suggested to signal nutrient receptor activation in the gastrointestinal tract [66]. While the direct influence of nutrients and hormones on the hypothalamus for controlling motivation towards food is well established in animals [67], the role of gastrointestinal nutrient receptors in humans is still under scrutiny. Intragastric infusion of glucose reduced appetite and concomitant food intake, bypassing the taste experience [68]; however, bypassing the gastrointestinal tract via direct venal infusion of glucose was not effective for reducing appetite versus saline infusion [69], suggesting a strong vagal influence via nutrient receptors [36]. In addition, oral glucose ingestion, but not intravenous infusion, increases GLP1 and GIP release, influencing insulin release and associated effects on the hypothalamus [70]. However, our study could not differentiate between vagal and direct influence of glucose and concomitant insulin changes on hypothalamic regulation of food motivation concerning negative feedback due to the fact that both pathways are activated after ingestion of sugar (ingestion trial).

Our study revealed that mouth rinse elevated food cravings and maintained levels post food cue exposure. Interstitial glucose was not elevated, showing that no sugar was accidentally swallowed. In addition, volume of water was ingested to compensate for potential activation of stretch receptor activation in the gastric cavity during the ingestion trial, when the same volume of sugary drink was swallowed [26]. Indeed, our data show that mere sensory activation, i.e., the experience of sweet taste, elevated food cravings when no nutrient-activated feedback was present, and levels were even higher after food cue exposure. Recent findings with sucralose investigating the effect on hypothalamic blood flow response [28] suggested that taste receptor activation without metabolic/hormonal feedback increases hypothalamic blood flow and the hunger response compared with sucrose intake, suggesting that noncaloric sweeteners might affect appetite regulation, representing a mismatch between sensory experience and metabolic feedback. While our study did not include noncaloric sweeteners, our results clearly suggest that food cravings are upregulated via oro-sensory and visual pathways in a feedforward manner, and, without negative feedback provided via sugar-dependent mechanisms projecting on hypothalamic pathways, craving is maintained. The outcomes reveal that a mismatch between sensory experience and a lack of nutrient/metabolic receipt results in increased experience of food cravings.

The importance of food cravings for eating behaviour is supported by many studies (e.g., see meta-analytic review by [20]). Moreover, ecological momentary assessment of food perception and eating behaviour using a phone application [71] revealed a positive correlation of trait craving levels with levels of perceived food wanting, which led to reported food intake. However, the study did not measure food-specific data. Diet diaries are prone to underreporting and inaccuracies [72,73], while the influence of keeping diet records on eating behaviour cannot be underestimated. For our study we used continuous glucose measurements to investigate the behavioural consequences of the distinct trials (ingestion or mouth rinse). A recent systematic review of 17 studies using continuous glucose measurement for automatic detection of food intake [74] concluded that automatic food intake detection is feasible based on the systems used, varying between 21% and 100% sensitivity. Larger increases in interstitial blood glucose are usually consequences of food intake; high-glycaemic index foods and larger amounts of intake will generate higher positive peaks in interstitial blood glucose, with inter-individual variability [56,75]. In our randomised-crossover design, inter-individual responses to the trials were noted; each participant performed both trials in random order with concomitant continuous measurement of interstitial glucose levels and calculation of the iAUC over a 10 h period after the trials. The outcomes revealed a significantly larger iAUC after the mouth rinse trial compared with the ingestion trial, which might suggest that the induced food cravings via taste perception and food cues without concomitant nutrient reception caused higher intake of carbohydrate/sugar foods in the following period. Food craving levels were positively correlated with iAUC in the rinse trial, supporting the former interpretation that maintenance of food craving levels after mouth rinse contributed to higher food intake. However, our iAUC is not a true measure of carbohydrate/food intake; therefore, the differences in iAUC might be caused by other mechanisms than food intake. However, these findings suggest the importance of sensory-metabolic mismatch for eating behaviour. Indeed, taste perception is modifiable through learning via former experience [76], and learned reward expectations in relation to taste and food cues drive motivation for food intake, as shown earlier [77,78]. The increase in food cravings after the combination of mouth rinse with food cues and the decrease in food craving after ingestion of sugar highlight the interaction of high-road and low-road regulatory connections [38] between gastrointestinal and blood nutrient sensory pathways (low road) and taste–visual pathways (high road) and suggest complex perceptual responses, as food cravings representing hedonic and homeostatic drives.

### 4.2. Moderating Physiological Mechanisms of Food Cravings

In our study, food cues robustly induced food cravings in the fasted state but not after sugar ingestion. This highlights the sensitivity to food cues during negative energy balance after the overnight fast, in agreement with others [15]. Moreover, an increase in food cravings was negatively associated with body fat, BMI, and leptin, revealing the moderating effect of body composition and leptin levels. Indeed, the negative correlation of leptin levels with food cue-induced craving increases revealed that individuals with lower leptin levels were more susceptible to food cues in the fasted state, suggesting an influence of leptin on the craving response to food cues. Leptin was shown to modify mesolimbic dopamine responsiveness to food cues [79,80]. Former work revealed that leptin levels were negatively associated with craving/hunger during energy restriction [81,82]. These outcomes could be interpreted as a differential influence of leptin with negative associations with food cue reactivity and a positive correlation of leptin with trait craving. Craving association with leptin was not seen after sugar intake, suggesting that glucose and insulin increases dominate acute negative feedback mechanisms towards the mesolimbic system, apart from other potential mechanisms (vagal, hormones). However, negative associations of leptin with iAUC were even seen after the ingestion trial, showing that medium-term adjustment of eating behaviour was still moderated by leptin levels after pre-load with sugar and concomitant homeostatic regulation. Moreover, associations of leptin with food cravings and iAUC were not detected after mouth rinse, showing that taste and food cues dominated the motivational drive towards food in relation to high-road regulation [38]. This notion is supported by the positive association of food cravings with iAUC after the rinse trial, suggesting that feedforward responses via taste and food cues may have influenced eating behaviour, encouraging subsequent intake.

Our study had several limitations. Ee did not measure brain activity during the experimental trials; therefore, the interpretations of our outcomes regarding a potential error signal produced by the combination of taste and food cues regarding food craving responses need to be further investigated by fMRI studies. Measurement of interstitial glucose iAUC is not a true measure of food intake; therefore, our results are only suggestive and need to be repeated with techniques that measure food intake directly. Moreover, our sample population did not include people with eating disorders and severe obesity; therefore, our findings cannot be extrapolated to those populations.

## 5. Conclusions

In conclusion, our study suggests that taste/flavour in connection with food cues generates an error signal which is experienced as food craving, and that the receipt of sugars with concomitant physiological responses leads to extinction of the signal and concomitant reduction in food craving. Taste/flavour in connection with food cues without receipt of nutrients seemed to motivate food intake in the following period, which suggests the importance of sensory-metabolic mismatch for food craving experience and eating behaviour.

## Figures and Tables

**Table 1:** Subjects’ anthropometric parameters and physiological characteristics.

Participants (n = 47; 30 Females)	Mean (SD)	Median [25th Percentile, 75th Percentile]
Age (years)	27.92 (7.95)	26.00 [23.0, 31.0]
Height (m)	1.70 (0.11)	1.65 [1.61, 1.76]
Weight (Kg)	67.56 (13.73)	67.70 [56.80, 78.20]
BMI (kg/m^2^)	23.89 (3.94)	23.43 [22.08, 25.74]
Estimated VO_2_ max (L × min^−1^ × kg^−1^)	42.10 (8.19)	39.92 [35.92, 49.47]
Body fat %	22.42 (8.23)	23.10 [16.20, 28.70]

Data are presented as means ± standard deviation (SD) or median and percentiles.

**Table 2:** Self-report measures and blood parameters.

Participants (n = 47; 30 Females)	Mean (SD)		Median [25th Percentile, 75th Percentile]
PANAS positive affect	36.09 (6.12)		37.00 [31.00, 41.00]
PANAS negative affect	20.15 (6.46)		19.00 [15.00, 24.00]
FCQ-T	120.38 (30.55)		123.00 [104.00, 133.00]
TFEQ: CR	53.84 (10.86)		52.78 [44.44, 63.89]
TFEQ: UE	48.86 (13.46)		51.85 [40.74, 59.26]
TFEQ: EE	58.39 (26.16)		55.56 [44.44, 77.78]
PSS	31.64 (3.76)		31.00 [29.00, 34.00]
IPAQ	High = 28	Mod = 16	Low = 3
Fasting glucose (mmol/L)	4.63 (0.47)		4.60 [4.30, 5.00]
Fasting leptin (ng/mL)	9.77 (9.75)		5.70 [3.17, 13.77]

Data are presented as means ± SD, median and percentiles; **PANAS (Positive)**: Positive and Negative Affect Schedule (Positive); Range: 10–50; higher scores representing higher levels of positive effect; **PANAS (Negative)**: Positive and Negative Affect Schedule (Negative); Range: 10–50; higher scores representing higher levels of negative affect; **IPAQ**: International Physical Activity Questionnaire; measured in MET minutes per week (MET minutes represent the amount of energy expended carrying out physical activity); **FCQ-T**: Food Craving Questionnaire-Trait; Range: 39–234. A higher score represents more frequent and intense food cravings; **TFEQ**: CR: Three-Factor Eating Questionnaire: Cognitive restraint; a higher score represents greater conscious restriction of food intake; **TFEQ**: UE: Three-Factor Eating Questionnaire: Uncontrolled eating; a higher score represents greater tendency to eat more than usual due to a loss of control over intake accompanied by subjective feelings of hunger; **TFEQ**: EE: Three-Factor Eating Questionnaire: Emotional eating; a higher score represents a greater tendency to overeat in response to negative emotions; **PSS**: Perceived Stress Scale; Range: 0–40; higher scores represent higher perceived stress. The mean age of the participants was 27.9 ± 8.0 years old, with a wide range of BMIs (17.30–35.50 kg/m^2^). The IPAQ reported the majority as having a moderate to high physical activity level (93%). The average estimated cardiovascular fitness level (VO_2_ max) of the participants was 42.10 ± 8.19 mL/kg/min.

**Table 3:** Interstitial glucose measurements over 14 days.

CGM Data	Mean (SD)	Median [25th Percentile, 75th Percentile]
Glucose variability (%CV)	16.06 (3.89)	16.00 [13.40, 18.70]
Mean glucose (mmol/L)	5.58 (3.89)	5.60 [5.30, 5.90]
Daytime glucose (mmol/L)	5.72 (0.42)	5.69 [5.47, 6.60]
Nighttime glucose (mmol/L)	5.31 (0.52)	5.41 [4.91, 5.70]

Data are presented as means ± SD; median and percentiles; glucose levels (mmol/L); glucose variability: percent coefficient of variation (%CV). CGM: Continuous Glucose Monitoring.

**Table 4:** Interstitial glucose levels.

Glucose Level	Fasted		
	Baseline	During FCQ-Ss	Change
Ingestion trial	**5.09 (0.81)** 5.20 [4.60, 5.60]	**5.07 (0.75)** 5.13 [4.60, 5.63]	**−0.02 (0.19)** −0.05 [−0.10, 0.08]
Rinse trial	**5.00 (0.64)** 5.10 [4.70, 5.50]	**5.02 (0.58)** 5.10 [4.70, 5.43]	**0.02 (0.15)** 0.03 [−0.03, 0.10]
Glucose level	After Intake		
	Baseline	During FCQ-S	Change
Ingestion trial	7.94 (1.41) * 8.10 [7.30, 9.10]	8.45 (1.38) * 8.63 [7.93, 9.33]	0.51 (0.46) * 0.53 [0.25, 0.80]
Rinse trial	**5.02 (0.63)** 5.10 [4.60, 5.50]	**5.04 (0.61)** 5.18 [4.58, 5.55]	**0.02 (0.09)** 0.00 [−0.08, 0.08]

Data are presented as means ± SD (bold); median and percentiles; glucose levels (mmol/L); * indicates significant change to baseline level in glucose levels *p* < 0.05; during FCQ-Ss: mean glucose during FCQ-S measurements.

**Table 5:** CGM data.

CGM Data		Mean (SD)	Median [25th Percentile, 75th Percentile]
Glucose iAUC (mmol*time)	Ingestion trial	28.98 (22.03)	30.70 [6.90, 44.60]
	Rinse trial	38.56 (16.21) *	37.80 [28.60, 52.50]

Data are presented as means ± SD; median and percentiles; glucose levels (mmol/L); glucose iAUC (mmol*time); * indicates significant difference between glucose iAUC after trials *p* < 0.05; glucose variability: percent coefficient of variation (%CV).

**Table 6:** FCQ-S craving scores based on standardised handgrip forces.

FCQ-S	Fasted		
	Pre-cue	Post-cue	Change
Ingestion trial	**6.28 (2.97)** 5.98 [3.78, 8.86]	**6.81 (3.15)** 7.54 [3.86, 9.41]	**0.53 (1.28)** * 0.66 [−0.32, 1.35]
Rinse trial	**6.38 (2.63)** 6.40 [4.44, 8.29]	**6.75 (2.64)** 6.86 [4.50, 8.85]	**0.37 (1.29)** # 0.42 [−0.55, 1.01]
FCQ-S	**After intake**		
	Pre cue	Post cue	Change
Ingestion trial	**6.30 (2.79)** 5.93 [4.32, 8.30]	**6.59 (2.94)** 6.30 [4.67, 9.20]	**0.26 (1.45)** 0.46 [−0.86, 1.42]
Rinse trial	**7.05 (2.95)** $\frac{*}{*}$ 7.25 [4.92, 9.67]	**7.24 (3.05)** 6.81 [4.77, 10.18]	**0.19 (1.49)** 0.17 [−0.91, 0.91]

Data is presented as means ± SD (bold); median and percentiles; **FCQ-S:** Food Craving Questionnaire-State scores (standardized units); * Indicates significant change in craving during ingestion trial when fasted *p* < 0.05; $\frac{*}{*}$ Indicates significant difference in craving between fasted and after intake for pre-cue time point during rinse trial *p* < 0.05; # indicates a trend towards significant change in craving during the rinse trial when fasted *p* < 0.10.

**Table 7:** Correlation analysis.

Variable 1	Variable 2	Correlation Coefficient (r)	*p*-Value
Craving change (fasted)	Body fat %	−0.371	0.010 **
Craving change (fasted)	BMI	−0.426	0.003 **
Craving change (fasted)	Leptin	−0.345	0.017 *
FCQ-T	Leptin	0.325	0.026 *
Fitness level	Body fat %	−0.710	<0.001 **
Fitness level	Leptin levels	−0.651	<0.001 **
Body fat %	BMI	0.515	<0.001 **
Body fat %	FCQ-T scores	0.295	0.044 *
Leptin levels	Body fat %	0.796	<0.001 **
iAUC (rinse trial)	Glucose variability	0.488	<0.001 **
iAUC (rinse trial)	Craving level (post cue)	0.321	0.03 *
iAUC (ingestion trial)	Leptin levels	−0.369	0.012 *

* Correlation is significant at the < 0.05 level (two-tailed); ** correlation is significant at the ≤ 0.01 level (two-tailed); Craving change: difference in craving between pre- and post-cue exposure; FCQ-T: Food Craving Questionnaire-Trait scores; iAUC: glucose iAUC; BMI: Body Mass Index.

## Data Availability

All data from this study are available at https://doi.org/10.5281/zenodo.17670127.

## Supplementary Materials

The following supporting information can be downloaded at: https://www.mdpi.com/article/10.3390/nu18010177/s1. Supplementary File: Food craving validation study [3,45,50,62,83,84,85,86,87,88,89,90,91,92,93]. Figure S1: Scatterplot showing the correlation between standardised scores of Food Cravings Questionnaire-state (FCQ-S) measured by the Likert scale, Y axis, and Handgrip forces, X axis; Figure S2: Bland-Altman plot for interrater agreement analysis. Table S1: Statements of the FCQ-S questionnaire; Table S2: Subject’s characteristics; Table S3: FCQ-S Likert scores in predicting nutritional state; Table S4: Significance of FCQ-S Likert scores in predicting state; Table S5: Handgrip scores in predicting nutritional state; Table S6: Significance of handgrip scores in predicting state; Table S7: Craving scores.
