# Supplementary material for: Taste of Things to Come: Craving Responses to Ingestion of and Mouth Rinse with a Sugary Drink in Connection with Food Cues and Associations with Continuous Interstitial Glucose Measurement in a Healthy Population
Source: Nutrients. 2026 Jan 5;18(1):177. doi: 10.3390/nu18010177 (PMC12787727; doi:10.3390/nu18010177)
Supplement: Supplementary file 1 [file nutrients-18-00177-s001.zip › nutrients-4029865-supplementary.pdf]

Supplementary File

Food Craving Validation Study

Introduction

Several self-reported questionnaires have assessed food craving, including the Food Craving Inventory (White, Whisenhunt, Williamson, Greenway, & Netemeyer, 2002), the Three-Factor Eating Questionnaire (Stunkard & Messick, 1985), and the state and trait Food Craving Questionnaires (FCQ) (Cepeda-Benito et al., 2000). The FCQs evaluate food craving as a complex concept with multiple dimensions on state (S) and trait (T) levels. The FCQ-S measures food cravings in the very moment across 5 dimensions using 15 items rated on a 7-point Likert scale. The FCQ-T assess trait craving with 39 items assessing seven food craving dimensions not influenced by acute changes, i.e., after food intake (Cepeda-Benito et al., 2000).

However, Likert scales may be prone to response bias. Especially when the scale is defined by expressions that serve as anchors, such as "strongly disagree" and "strongly agree", with responses leading to skewed or polarised distributions (Jamieson, 2004). The use of a 5 or 7-point Likert scale may result in respondents selecting the middle point of the scale (e.g., "neither agree nor disagree") instead of more extreme values, known as "central tendency bias", and other key response biases like acquiescence bias and social desirability bias (Jebb, Ng, & Tay, 2021; Westland, 2022). Overall, studies imply the possibility of response bias using Likert scales and alternative methods of assessment of food cravings should be sought.

To reduce response bias, handgrip force might be used as a surrogate measure for the level of agreement to questionnaire statements. This entails using a handgrip dynamometer for the force exerted by an individual squeezing the handle (Harkonen, Harju, & Alaranta, 1993). Handgrip strength is consistent over extended periods of time, taken over weeks, months, or even years (Shechtman, Gestewitz, & Kimble, 2005). As a psychometric measure, handgrip force was used as a measure of ‘visceral’ response to language stimuli (Thoma, Hüsam, & Wielscher, 2023). In context with eating behaviour, Arumäe et al. (Arumäe, Kreegipuu, & Vainik, 2019) used handgrip force for grading explicit food wanting and liking responses and demonstrated its construct validity.

Concurrently, the objectives of this study were to validate the use of handgrip force as a response for the FCQ-S items instead of Likert scale responses (Cepeda-Benito et al., 2000). In addition, we aimed to investigate which measure (Likert scale or handgrip force) was a better predictor of fasted and postprandial states of participants. Craving levels are reported to be affected by food intake (Cameron, Goldfield, Finlayson, Blundell, & Doucet, 2014) (Cepeda-Benito et al., 2000; Nederkoorn, Smulders, & Jansen, 2000). Therefore, this study also evaluated the craving level pre- and post-food intake using both assessment methods for comparison.

We hypothesised that handgrip force is a valid construct for responses to FCQ-S statements, with greater predictive accuracy for distinguishing between fasted and postprandial states than the Likert scale.

Methods

Ethical approval:

This study was ethically approved by the Ethics Committee of School of Psychology and Sport Sciences at Bangor University, ethics number: **2022-17200**.

Participants:

32 healthy participants (18 males and 14 females) were recruited from Bangor University and the local community in the Bangor area. Participant inclusion criteria were aged 18-45 years old, no injuries which might affect handgrip force, not pregnant, non-smoker, healthy, i.e., without any cardiovascular, metabolic, or pulmonary disease, BMI between (18 to 35 kg/m^2), no medications which might limit participation, and absence of dietary requirements including allergies or intolerances of diary, not being vegan (due to test meal). Participants consented after reading the participant information sheet and after assessing eligibility. Participants received a reimbursement of £10 for their time after a successful completion of the study.

Procedures:

The study consisted of one session divided into two phases (fasted and after food intake). Study material was integrated into Qualtrics (www.qualtrics.com). Participants performed the session

after an overnight fast, having refrained from strenuous exercise and alcohol in the preceding 48 hours. The session consisted of the following sections:

Baseline data was collected, including sex, age, height, and weight.

Familiarisation with the handgrip and FCQ-S - Participants were familiarised with the FCQ-S and using handgrip force to represent their agreement with the statements of the Food Craving Questionnaire State (FCQ-S), as well as introducing them to the Likert scale-based assessment. After explaining and familiarization with measures, three maximum handgrip forces for the standardisation of cravings were recorded. A handgrip dynamometer was used to measure forces connected to an A/D converter (PowerLab system).

Testing – After an overnight fast, the participants were either asked to rate their current cravings (FCQ-S statements) by applying handgrip forces for perceived agreement with statements or with the Likert scale. The order of two sets of the FCQ-S items (Table S1) (handgrip and Likert scale) was randomised, and items were depicted on a computer screen and responses were stored on Qualtrics (Likert response) and Powerlab PC (handgrip force). Food Cravings Questionnaire-State (FCQ-S) uses 15 items measuring the agreement with the statements connected to 5 dimensions, including (an intense desire to eat; anticipation of positive reinforcement that may result from eating; anticipation of relief from negative states and feelings as a result of eating; lack of control over eating and craving as a physiological state). The FCQ-S has an overall CRα of 0.96 (Cepeda-Benito et al., 2000). Consequently, 30 randomised statements, 15 answered with a Likert scale from 1 (strongly disagree) to 7 (strongly agree), and 15 statements were to be responded to by handgrip force, a diagram which showed the scale with the anchor words on the screen (minimal force = strongly disagree, maximum force = strongly agree). The craving response by the handgrip (HG) was standardised using: (test HG value – minimum HG value) / (maximum HG value – minimum HG value), minimum value being the value by just holding the dynamometer and maximum being the highest value produced for the maximum handgrip test during familiarisation session.

Table S1: statements of the FCQ-S questionnaire.

I have an intense desire to eat something tasty.
I am craving food.
I have an urge for food.
Eating food would make things just perfect.

If I were to eat what I am craving, I am sure my mood would improve.
Eating food would feel wonderful.
If I ate something, I would not feel so sluggish and lethargic.
Satisfying my craving would make me feel less grouchy and irritable.
I would feel more alert if I could satisfy my craving.
If I had food, I would not stop eating it.
My desire to eat food seems overpowering.
I know I am going to keep on thinking about food until I actually have it.
I am hungry.
If I ate right now, my stomach would not feel as empty.
I feel week because of not eating.

In the second part of the testing, participants consumed the test meal (Resource® Energy Vanilla 200ml, Nestlé, Switzerland) and rested for 20 minutes before reiteration of the craving assessments as before.

Statistical Analysis

All statistical analyses were performed using IBM SPSS Statistics 29 and Microsoft Excel. All data are reported as means ± SD, unless otherwise stated. Statistical significance was set at $P<0.05$. All data were assessed for relevant assumptions: normality, outliers, and homogeneity of covariance. Handgrip forces and FCQ-S data were square root transformed to normality. Pearson's correlation was used to analyse relationships between standardised handgrip values and FCQ-S Likert scores. A binary logistic regression analysis was employed to ascertain which variable more effectively predicts the physiological state, either fasted or postprandial. A one-way analysis of variance (ANOVA) was conducted to evaluate changes in craving levels before and after food intake. Subsequently, to further examine the agreement between the two methods of measuring cravings on the FCQ-S, a Bland-Altman plot was constructed (Bland & Altman, 1999). In the plot, the X axis shows the mean score across both measures, and the Y axis shows the difference between the two measures.

Results

Table S2 depicts the body characteristics of the participants of the sample.

Table S2: Subject's characteristics

Participants (n=32; 14 females)	Mean (SD)
Age (years)	24.66 (7.34)
Height (m)	1.71 (0.10)
Weight (Kg)	72.23 (15.55)
BMI (kg/m2)	24.49 (4.34)

The relationship between FCQ-S Likert scores and handgrip scores for food cravings:

The relationship between the two types of craving measures (FCQ-S Likert scale and Handgrip forces) was assessed by Pearson's correlation analysis. The results revealed a statistically significant positive correlation between the two measures ($r = 0.689$, $p < .001$), suggesting that the two assessment methods are capturing related conceptual measures for assessing the craving construct (Figure S1).

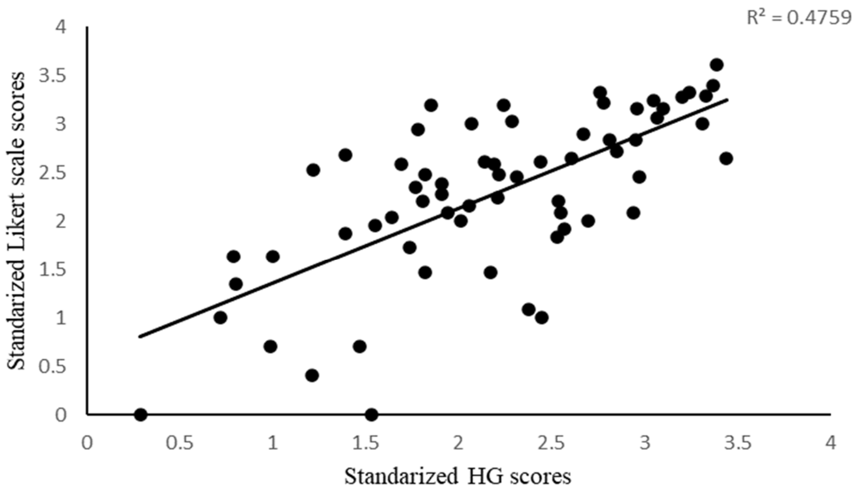

***Figure S1**: Scatterplot showing the correlation between standardised scores of Food Cravings Questionnaire-state (FCQ-S) measured by the Likert scale, Y axis, and Handgrip forces, X axis.*

Furthermore, agreement between the Likert scale and handgrip force measures of craving response was assessed using a Bland-Altman plot (Figure S2). The plot revealed a mean difference of 0.0833 (SD = 0.6446), with most data points falling within the 95% limits of agreement, indicating reasonable concordance between the two methods. However, a slight positive bias and a few outliers were noted. Moreover, a one-sample t-test was conducted to determine whether the mean difference between the standardised Likert scale and handgrip force scores significantly differed from zero. The results showed a non-significant difference

($t(63) = 1.050$, $p = .298$, two-tailed), showing that the two measures, on average, yield similar craving response scores. In summary, while the Bland-Altman plot suggests generally good concordance between the two methods with a few outliers, the non-significant t-test confirms that the two measures produce comparable scores overall for quantifying craving response.

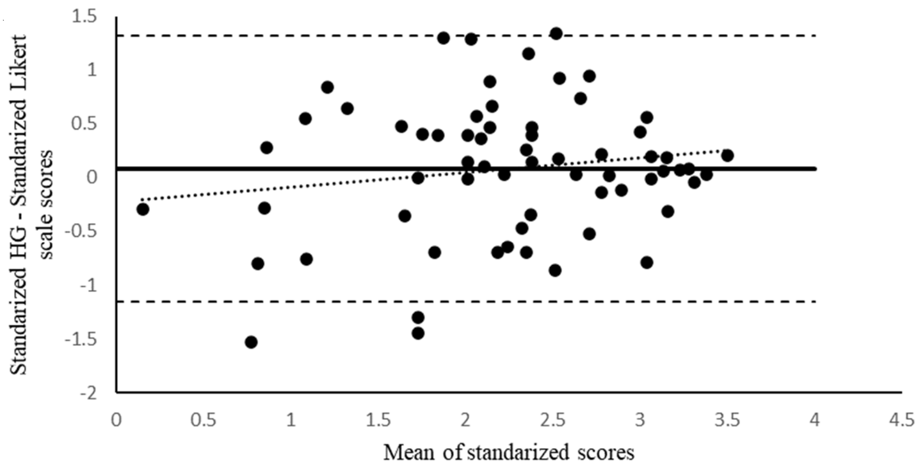

Figure S2: *Bland–Altman plot for interrater agreement analysis; Limits of Agreement are shown as dashed, black lines with 95% confidence intervals; bias as solid black line; and regression fit of the differences on the means as circle dotted black line.*

Prediction of fasted and postprandial states

To determine which assessment method better predicts the state (fasted and postprandial) of the participants, a binary logistic regression analysis was conducted comparing the standardised FCQ-S Likert scale scores and the standardised handgrip (HG) scores in fasted and postprandial states.

Logistic regression analysis showed that handgrip forces were better at predicting the state (fasted/postprandial) compared with the FCQ-S Likert scale responses; handgrip forces predicted 75% ($p = .001$) of the state correctly, while the FCQ-S Likert scale responses only 57.8% ($p = .044$). This finding suggests that handgrip force measurements may be a more reliable and accurate method for assessing food cravings using the FCQ-S items (Tables S3, S4, S5, and S6).

Table S3: FCQ-S Likert scores in predicting nutritional state.

Observed State	Predicted as Fasted	Predicted as Postprandial	Percentage Correct
**Fasted**	20	12	62.5%
**Postprandial**	15	17	53.1%

Overall Percentage		57.8%

Table S4: Significance of FCQ-S Likert scores in predicting state.

Variable	B	S.E.	Wald	df	Exp(B)	Sig.
**Standardised FCQ-S Likert Scores**	-0.677	0.336	4.049	1	0.508	.044 *
**Constant**	1.557	0.825	3.560	1	4.746	.059

** Indicates the significance < 0.05 level.*

Table S5: Handgrip scores in predicting nutritional state.

Observed State	Predicted as Fasted	Predicted as Postprandial	Percentage Correct
**Fasted**	24	8	75.0%
**Postprandial**	8	24	75.0%
**Overall Percentage**			75.0%

Table S6: Significance of handgrip scores in predicting state.

Variable	B	S.E.	Wald	df	Exp(B)	Sig.
**Standardised Handgrip Scores**	-1.401	.438	10.245	1	.246	.001 **
**Constant**	3.106	1.018	9.313	1	22.327	.002

*** Indicates the significance ≤ 0.01 level.*

Changes in food craving scores:

Changes in craving scores by the FCQ-S Likert scale and handgrip were assessed during both fasted and postprandial states. The craving scores were transformed for normality, followed by one-way ANOVA revealing a significant effect of state on craving scores measured by the Likert scale, $F(1, 62) = 4.545$, $p = .037$, indicating that the state significantly affected self-reported craving scores. A more pronounced effect was observed for the handgrip force measurements, with a significant state effect, $F(1, 62) = 14.534$, $p < .001$. (Table S7). The craving assessment via handgrip force measurement demonstrated a stronger ability to detect changes in craving scores between the fasted and postprandial states compared to the Likert scale.

Table S7. Craving scores.

	Fasted	Postprandial
**FCQ-S Likert scale scores**	**2.50 (0.68)**<br>2.61 [1.97,3.16]	**2.07 (0.94) ***<br>2.25 [1.51, 2.67]
**Handgrip scores**	**2.53 (0.59)**<br>2.56 [2.18, 2.96]	**1.88 (0.76) ****<br>1.91 [1.39, 2.22]

Data is presented as means ± SD (bold); median and percentiles; ***FCQ-S:*** *Food Craving Questionnaire-State scores (normalized units); * Indicates significance change in craving p<0.05; ** Indicates significance change in craving p<0.001.*

Discussion

The present study aimed to validate the use of handgrip force (HG) as a measure of the FCQ-S. Additionally, the study investigated whether HG forces would be a better predictor of the participants' state (fasted/satiated) compared to the FCQ-S Likert scale. Furthermore, craving levels were assessed before and after food intake using both measurement methods.

The study demonstrated significant moderate to strong positive correlations between the FCQ-S Likert and handgrip force scores, supporting the first hypothesis that the two measures of craving would measure related constructs. These results are consistent with previous research by Arumäe et al. (Arumäe et al., 2019), which investigated the construct validity of handgrip force for explicit wanting and liking of food. However, the Bland-Altman plot revealed a bias between the two measures, indicating that they do not agree. However, using logistic regression analysis, the handgrip force predicted the state of fasted and postprandial substantially better than the Likert scale. Previous studies have confirmed that Likert scores show increased craving levels in fasted states compared to giving a test meal, leading to decreased craving levels (Cameron et al., 2014; Rogers & Hardman, 2015). This is the first known study to assess the use of handgrip force for food cravings using FCQ-S. However, Crestwell et al. (Creswell et al., 2019) investigated the handgrip force measure of cravings in comparison to verbally reported craving scores on a 0-100 scale measuring cigarette cravings in smokers. They demonstrated that using the handgrip to measure grip strength was a reliable and valid method for assessing cravings in smokers.

In conclusion, the present study reveals that the handgrip measure reports a much better prediction of the nutritional state of participants, as well as reflecting a higher sensitivity in assessing differences in food cravings compared with the FCQ-S Likert scale use.

References

Arumäe, K., Kreegipuu, K., & Vainik, U. (2019). Assessing the overlap between three measures of food reward. *Frontiers in Psychology, 10*, 883.

Bland, J. M., & Altman, D. G. (1999). Measuring agreement in method comparison studies. *Statistical Methods in Medical Research, 8*(2), 135-160. doi:10.1177/096228029900800204

Cameron, J. D., Goldfield, G. S., Finlayson, G., Blundell, J. E., & Doucet, E. (2014). Fasting for 24 hours heightens reward from food and food-related cues. *PLoS One, 9*(1), e85970. doi:10.1371/journal.pone.0085970

Cepeda-Benito, A., Gleaves, D. H., Fernandez, M. C., Vila, J., Williams, T. L., & Reynoso, J. (2000). The development and validation of Spanish versions of the State and Trait Food Cravings Questionnaires. *Behav Res Ther, 38*(11), 1125-1138. doi:10.1016/s0005-7967(99)00141-2

Creswell, K. G., Sayette, M. A., Skrzynski, C. J., Wright, A. G., Schooler, J. W., & Sehic, E. (2019). Assessing cigarette craving with a squeeze. *Clinical Psychological Science, 7*(3), 597-611.

Harkonen, R., Harju, R., & Alaranta, H. (1993). Accuracy of the Jamar dynamometer. *J Hand Ther, 6*(4), 259-262. doi:10.1016/s0894-1130(12)80326-7

Jamieson, S. (2004). Likert scales: how to (ab)use them. *Med Educ, 38*(12), 1217-1218. doi:10.1111/j.1365-2929.2004.02012.x

Jebb, A. T., Ng, V., & Tay, L. (2021). A Review of Key Likert Scale Development Advances: 1995-2019. *Front Psychol, 12,* 637547. doi:10.3389/fpsyg.2021.637547

Nederkoorn, C., Smulders, F. T., & Jansen, A. (2000). Cephalic phase responses, craving and food intake in normal subjects. *Appetite, 35*(1), 45-55. doi:10.1006/appe.2000.0328

Rogers, P. J., & Hardman, C. A. (2015). Food reward. What it is and how to measure it. *Appetite, 90*, 1-15.

Shechtman, O., Gestewitz, L., & Kimble, C. (2005). Reliability and validity of the DynEx dynamometer. *J Hand Ther, 18*(3), 339-347. doi:10.1197/j.jht.2005.04.002

Stunkard, A. J., & Messick, S. (1985). The three-factor eating questionnaire to measure dietary restraint, disinhibition and hunger. *J Psychosom Res, 29*(1), 71-83. doi:10.1016/0022-3999(85)90010-8

Thoma, D., Hüsam, J., & Wielscher, K. (2023). Introducing grip force as a nonverbal measure of bilingual feelings. *Bilingualism: Language and cognition, 26*(1), 125-137.

Westland, J. C. (2022). Information loss and bias in likert survey responses. *PLoS One, 17*(7), e0271949. doi:10.1371/journal.pone.0271949

White, M. A., Whisenhunt, B. L., Williamson, D. A., Greenway, F. L., & Netemeyer, R. G. (2002). Development and validation of the food-craving inventory. *Obes Res, 10*(2), 107-114. doi:10.1038/oby.2002.17
